# Extracellular Vesicles Are Associated With Biological, Mechanical, and Structural Tendon Healing Metrics in Preclinical Models: A Systematic Review and Meta‐Analysis

**DOI:** 10.1002/jor.70289

**Published:** 2026-10-08

**Authors:** Gabrielle Gilmer, Kariman Shama, Liam Klingensmith, Jesse Zhang, Brittany Taylor, Allison C. Bean

**Affiliations:** ^1^ Medical Scientist Training Program University of Pittsburgh School of Medicine Pittsburgh Pennsylvania USA; ^2^ Department of Biomedical Engineering University of Florida Gainesville Florida USA; ^3^ Bethel Musculoskeletal Research Center University of Pittsburgh Pittsburgh Pennsylvania USA; ^4^ Department of Bioengineering Swanson School of Engineering, University of Pittsburgh Pittsburgh Pennsylvania USA; ^5^ Department of Pharmacology and Therapeutics University of Florida Gainesville Florida USA; ^6^ Department of Physical Medicine and Rehabilitation University of Pittsburgh School of Medicine Pittsburgh Pennsylvania USA; ^7^ McGowan Institute for Regenerative Medicine University of Pittsburgh School of Medicine Pittsburgh Pennsylvania USA

**Keywords:** extracellular vesicles, orthobiologics, preclinical models, systematic review, tendon healing

## Abstract

Tendinopathies comprise half of musculoskeletal injuries, yet medical treatments are lacking. Orthobiologics are novel therapeutics developed to address this gap, with extracellular vesicles (EVs) being a promising prospect. However, we lack a complete understanding of how EVs affect regeneration, both endogenously and as a tool for augmenting tendon healing. Therefore, the purpose of this review was to compile the literature focused on understanding the impact of EV treatment on tendon healing using animal models. We performed a literature search in accordance with PRISMA reporting guidelines. Title, abstract, and full‐text screening were performed by two authors. We assessed transparency and risk‐of‐bias using the ARRIVE, MISEV, and SYRCLE Guidelines, and meta‐analyses were performed on overlapping outcome variables. After screening, 46 articles were included. Most studies utilized young male Achilles tendons, with a diversity of EV cell sources, injury types, dosage schemes, and biomaterials. Several ARRIVE (e.g., blinding) and MISEV (e.g., seeding density) recommendations remain poorly reported in this literature, and the risk of bias per SYRCLE guidelines is relatively high. Despite high heterogeneity, EV treatment was associated with improved histological tendon healing scores and increased load to failure, tendon stiffness, and Young's modulus. Further meta‐analyses revealed EV treatment was associated with increased type I collagen protein and type III collagen transcript expression, and decreased protein expression of IL‐6 and markers of senescence. We recommend that future studies include different ages, sexes, and more systematically protocolized EV delivery scheme.

**Statement of Clinical Significance:** This systematic review identifies gaps in the tendon and EV literature while also unveiling unexplored mechanisms by which EVs may promote tendon healing. This improved understanding and call for rigor in studies enhances the translational potential of EVs as a future treatment for tendon injuries.

## 1 Introduction

Approximately 50% of musculoskeletal injuries involve tendons [1, 2]. Specifically, the incidence of acute tendon ruptures in the United States is estimated to be 3.80 per 100,000 person‐years [3], and 22% of adults over the age of 60 are estimated to have chronic tendon tears [4, 5]. Currently, activity modification, physical therapy, and anti‐inflammatory medications are the mainstays of non‐surgical interventions [6]. However, 10%–45% of tendinopathies fail conservative treatment [6]. Of these patients, 29%–42% ultimately have surgical intervention [7], with re‐tear rates up to 94% [8, 9]. For patients who opt for non‐surgical management, 35%–50% may continue to have chronic symptoms [10, 11, 12]. Thus, there is a significant need for improved medical treatment of tendon pathology.

Over the last two decades, clinicians and scientists have explored novel orthobiologic interventions for treating tendinopathies. Currently, platelet rich plasma (PRP) [13], bone marrow aspirate concentrate (BMAC), micro‐fragmented adipose tissue (M‐FAT) [14], and collagen patch augmentation are increasingly utilized in the clinical settings [15]. However, the efficacy of such treatments has been challenging to determine as we lack an understanding of how these orthobiologics contribute to tendon healing, if at all. These shortcomings are due, at least in part, to research studies being consistently plagued by heterogenous design, inconsistent outcomes, and incomplete reporting [13, 15].

Extracellular vesicles (EVs) have the potential to both close the gap in our mechanistic understanding of native tendon regeneration and provide an orthobiologic tool to enhance healing. EVs are nanometer to micrometer sized membranous structures containing nucleic acids, proteins, and lipids [16]. They are secreted by nearly all cells and tissues and have increasingly been shown to play a role in healing through recruiting inflammatory cells to the injury site, modulating resident cell phenotype, increasing proliferation, and promoting tissue repair [17]. As an acellular biologic, EVs have low immunogenicity, may have better stability in long term storage, and can be engineered to stimulate cell specific reactions [18, 19], suggesting they may enable more individualized, precision medicine therapeutic strategies in comparison to current cell‐based approaches [20].

However, similar to other orthobiologics, high variability in experimental design among pre‐clinical studies remains a consistent challenge. Donor source, isolation and characterization methods, as well as dosage and frequency of EV administration differ widely, in addition to the tendon and injury model utilized. In the last 5 years, several reviews have evaluated how EVs modulate tendon‐bone incorporation [21], Achilles tendon healing [22], and the tendon immune environment [23]. Systematic reviews of the pre‐clinical literature focused on understanding how EVs impact tendon healing are limited [21, 22, 24], none have utilized meta‐analyses to identify possible mechanisms, and assessment of transparency in reporting have been limited. Therefore, our goal was to systematically review the published literature, quantify transparency in reporting and risk‐of‐bias among said literature using published guidelines, and meta‐analyze how EV administration impacts tendon healing following injury using in vivo animal models. In line with the 2023 Minimal Information for Studies of Extracellular Vesicles (MISEV) reporting guidelines, we define EVs as particles naturally released from cells, delimited by a lipid bilayer, and unable to replicate independently [25]. This generic, umbrella term includes exosomes, small EVs (< 200 nm), medium/large EVs (> 200 nm), and apoptotic vesicles.

## 2 Methods

### 2.1 Systematic Review

We performed a systematic review of the literature in accordance with the Preferred Reporting Items for Systematic Reviews and Meta‐Analyses (PRISMA) reporting guidelines (Appendix S1) [26], Meta‐Analysis of Observational Studies in Epidemiology (MOOSE) checklist (Appendix S2) [27], Cochrane Handbook for Systematic Reviews of Interventions [28], and the practical guide for meta‐analysis in animal studies [29]. Although we did not register this systematic review on PROSPERO *a priori* [30], we followed a pre‐specified protocol for inclusion and exclusion of articles, as well as for our analyses. We identified eligible manuscripts using the following PECO [31]: Population–Animals (no species, age, or sex limitation), Exposure—EV treatment following an injury (no limitation on type of native EV, where the EVs came from, or injury type), Comparison—no EV treatment following an injury (age/sex/injury matched control), and Outcome–metrics of tendon health and healing. Specific search terms are listed in Table S1. Studies were excluded if they were written in a non‐English language, not peer reviewed, not original research articles, did not include age/sex matched controls, there was no tendon injury performed in either group, or the “injury” was an infection. There was no exclusion based on the year of publication. Studies that exclusively included engineered EVs (e.g., the experimental group was engineered EVs and the control group were native EVs) were excluded. If a study contained a native EV, an engineered EV group, and a control, only the native EV group was included as an experimental group. We elected to exclude engineered EVs from this analysis to ensure comparability of intrinsic EV bioactivity across studies.

On April 13, 2026, we performed a literature search on PubMed, Physiotherapy Evidence Database (PEDro), EBSCO, Web of Science, Scopus, and Cochrane Central Register of Controlled Trials in attempt to exhaustively collect all preclinical articles on this topic. Two authors independently screened the titles, abstracts, and full texts of studies identified via our search based on the aforementioned PECO and Cochrane Handbook recommendations [28]. Reasons for exclusion were documented at the full text level (Table S2). Following full text screening, we performed a citation search via PubMed to identify additional publications for screening. Screening was completed in pre‐designed spreadsheets, and disagreements between authors were adjudicated by the opinion of a third author. For the citation search, articles that cited the identified and included articles at the full text level were extracted, and title, abstract, and full text screening were repeated as described above.

Two authors collected study demographic information from included articles (Table S3), and one author extracted data for use in meta‐analyses. Graphical data were transcribed to numerical data using a validated digital ruler that has been previously reported to have ‘excellent’ intra‐rater reliability for graphical to numerical data conversion under our use (*n* = 5, intraclass correlation coefficient: 0.9752, 95% CI: [0.9413, 1.0000]) [32, 33]. We further validated independent measurements performed by two authors and detected ‘excellent’ inter‐rater reliability (*n* = 15, interclass correlation coefficient: 0.997, 95% CI: [0.990, 0.999]).

#### 2.1.1 Reporting Quality, EV‐Reporting Transparency, and SYRCLE Risk of Bias assessments

Two authors independently assessed the reporting quality of the included studies using the full Animal Research: Reporting In Vivo Experiments (ARRIVE) Guidelines 2.0 [34]. The 21 categories were ranked as either “clearly insufficient” (0), “unclear if sufficient” (1), or “clearly sufficient” (2). The highest possible score of 42 indicates more transparent reporting, while a 0 indicates no transparency. Scores between the two authors were averaged. Spearman's correlation analysis between ARRIVE score and year of article publication was calculated via SPSS Statistics for Windows, version 31 (IBM Corp., Armonk, NY, USA).

Additionally, two independent authors used the Minimal Information for Studies of Extracellular Vesicles (MISEV) Guidelines to evaluate transparency in reporting of key EV isolation methods and characteristics [25, 35, 36]. Categories were treated as binary, with a 1 indicating that category was reported and a 0 indicating that category was not reported. Similar to the ARRIVE scores, we correlated the total number of reported items with year of publication using a Spearman correlation analysis in SPSS Statistics. In our supplementary table, we include guidelines from all three versions (2014, 2018, 2024), but in our figure and analyses, we only include guidelines from 2018, given that all papers were published after this date and had the opportunity to ensure compliance with the guidelines.

Lastly, two authors independently assessed the SYstematic Review Centre for Laboratory animal Experimentation (SYRCLE) risk of bias (ROB) in publications guidelines [37]. The ten categories were ranked as either “high risk” (0), “unclear risk” (1), or “low risk” (2). The highest possible score of 20 indicates the lowest risk of bias, while 0 indicates a relatively high risk of bias. Scores between the two authors were averaged. Spearman's correlation analysis between SYRCLE ROB score and year of article publication was calculated via SPSS Statistics. We note that while the validated SYRCLE uses scoring of high risk/unclear risk/low risk, the conversion of this to a numerical scale and ultimate use in a correlation analysis is not formally validated and is used here as an exploratory analysis.

### 2.2 Dose Response Curve and Visualization

In an attempt to examine the effect of dose on histological measure of tendon healing, we visualized the relationship between dose with the fixed effect size of histological outcome of tendon healing. Manuscripts reported EV doses using two different methods: protein in micrograms (µg) or particle number, normalized to animal weight and volume of injection. Therefore, we visualized two relationships: one including studies that reported EV dose using EV protein mass and another including studies that reported number of EV particles administered. We plotted inverse variance as the marker size to provide insight into study reliability. Only studies in which one dose of EVs was administered immediately after the injury were included in this visualization to reduce the number of confounding variables.

### 2.3 Meta‐Analyses

We performed meta‐analyses on histological scores of tendon healing, maximum load to failure, stiffness, Young's modulus, type I & III collagen protein and transcript expression, IL‐6 protein expression, and protein markers of senescence (p16, p21, senescence‐associated beta‐galactosidase (SA‐β‐GAL)). These outcomes were selected as they were the only ones with more than four independent studies with quantification. If a study reported multiple outcomes (e.g., a study reported histological score based on H&E and quantified load to failure), those outcomes were included in both analyses. We calculated the standardized mean differences (SMD) and pooled standard deviations (SD_pool_) of these variables from included studies via the DerSimonian‐Laird method, which accounts for between‐study heterogeneity under a random‐effects framework and permits pooling across studies using different outcome measures and scoring systems [38]. We chose these specific methods because the outcomes assessed in our meta‐analyses include comparable outcomes with different measurement scales. We selected a random‐effects model using the DerSimonian‐Laird estimator to account for anticipated between‐study heterogeneity. For histological scoring, we oriented all scores such that a higher score indicates higher quality tendon healing, and a lower score indicates more pathology. For studies with multiple time points and multiple treatment dosages, the time point furthest out from injury and/or the highest dose were included. For studies with multiple treatment arms, only native EVs were utilized in our analyses (i.e., no engineered EVs). If studies had a native, manipulated, and a control group, only the native group was included in our analysis. Cochran's Q test was utilized to assess heterogeneity and homogeneity, and statistical significance was defined as an alpha level of 0.05. Meta‐analyses with random effects were also performed via SPSS Statistics for Windows, version 31 with no small‐sample correction added. We chose not to include a small‐sample correction, given that the sample sizes varied from small to moderate to large samples. Specifically, mean, sample size, and standard deviation from the original studies were input into SPSS to calculate the effect size, 95% confidence interval, and weight of each study within the meta‐analysis.

## 3 Results

### 3.1 Wide Variability in EV Origin, Characterization, and In Vivo Injury Models

Following database searches, we identified 344 unique studies related to EV exposure and tendon healing following an injury. After title, abstract, and full text screening, 42 studies were considered eligible for inclusion in our review. After a citation search, we identified four additional studies for inclusion, leading to 46 studies being included in our analyses (Figure 1) [39, 40, 41, 42, 43, 44, 45, 46, 47, 48, 49, 50, 51, 52, 53, 54, 55, 56, 57, 58, 59, 60, 61, 62, 63, 64, 65, 66, 67, 68, 69, 70, 71, 72, 73, 74, 75, 76, 77, 78, 79, 80, 81, 82, 83, 84].

**Figure 1:**
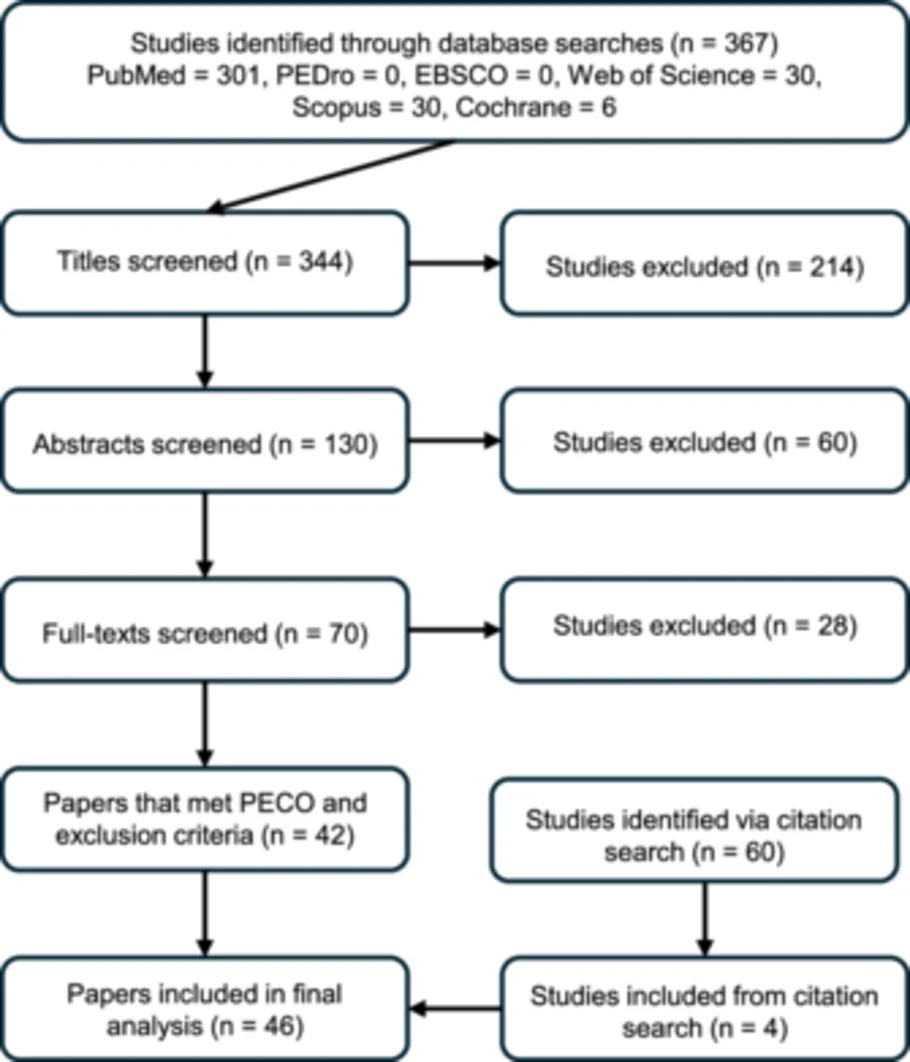
Systematic review workflow**.** After title, abstract, and full text screening as well as a citation search, 46 articles were determined to be eligible for inclusion in this review. Reasons for exclusion at the full text level are reported in Table S2 and basic study information about included articles is listed in Table S3.

Eighteen studies (39%) isolated EVs from rat cells (Figure 2A), with bone marrow derived and adipose stem cells being the most frequent cell type (*n* = 10 & 11, 22 & 24% respectively, Figure 2B). Most studies utilized cells from passages 3–5 (*n* = 16–21, 35%–46%), while thirteen studies (27%) did not report what cell passage was utilized (Figure 2C). Thirty studies (65%) used EVs from a young cell source, while 13 studies (28%) did not report the age of the cells utilized (Figure 2D). Similarly, 23 studies (50%) utilized only male cells to generate their EVs, and 16 studies (35%) did not report sex (Figure 2E). CD63 (*n* = 24, 52%), CD9 (*n* = 26, 57%), and TSG101 (*n* = 22, 48%) were the most common markers utilized for EV characterization (Figure 2F). Notably, few studies reported markers to ensure purity of EV samples (e.g., ApoA1, ApoB), as recommended by the MISEV Guidelines [25]. Most studies included EVs that ranged from 100 to 300 nm in diameter (*n* = 28–45, 61%–98%) (Figure 2G).

**Figure 2:**
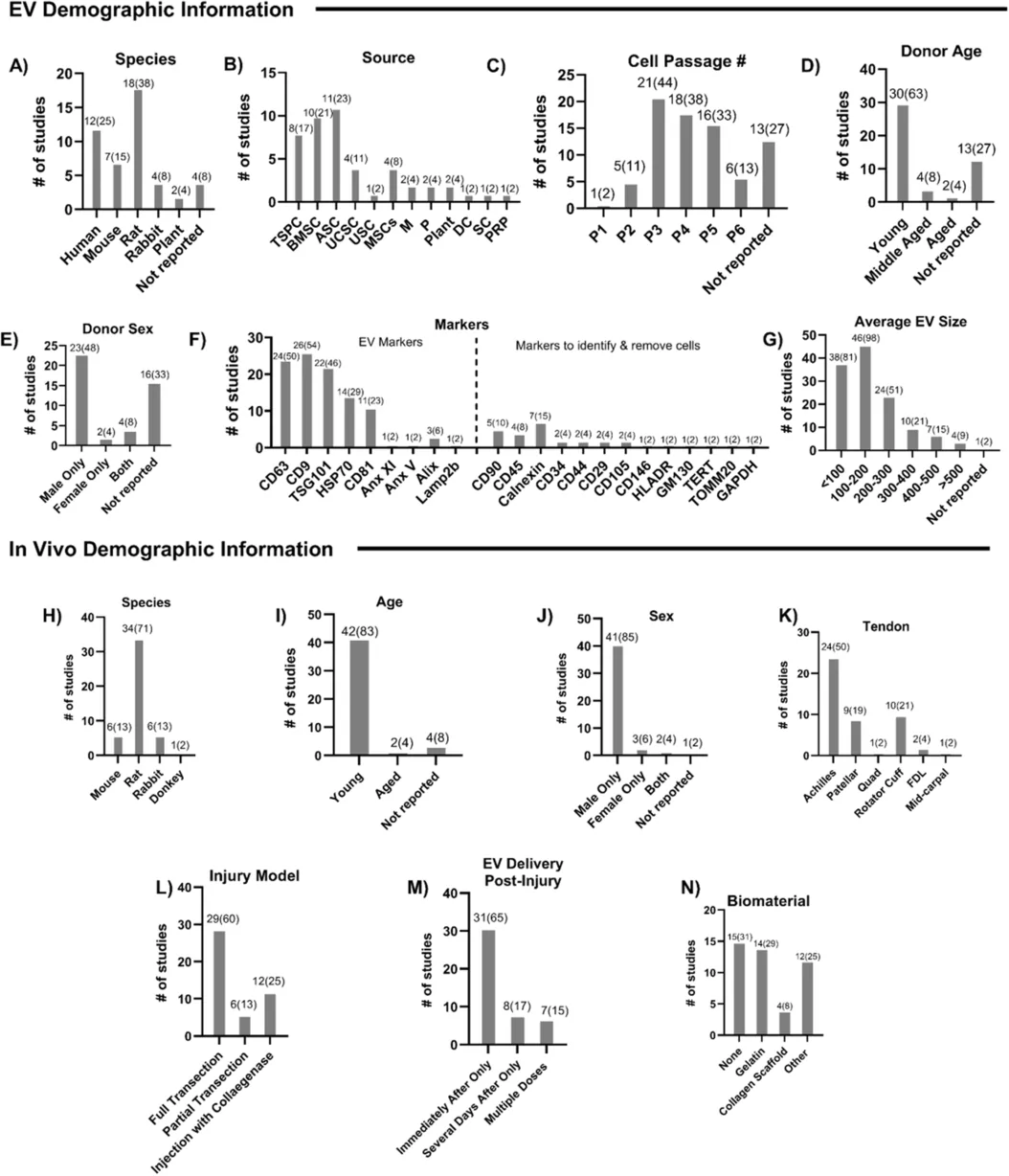
Extracellular vesicle (EV) and in vivo demographic information from included studies. Numbers over bars are # of studies (% of studies). (A) EV Species. (B) EV Source. Adipose stem/stromal cells (ASC). Bone marrow derived stem/stromal cells (BMSC). Dendritic cells (DC). Mesenchymal stem cells (MSCs) derived from induced pluripotent stem cells (iMSC). Macrophages (M). Platelets (P). Tendon stem/progenitor cells (TSPC). Umbilical cord stem cells (UCSC). Urine stem cells (USC). (C) Passage number of cells that produced EVs. (D) Age of cell that produced EVs. (E) Sex of cell that produced EVs. (F) Markers used to categorize EVs. (G) EV Size included in study. (H) Species of in vivo animal model. (I) Age of in vivo animal model. (J) Sex of in vivo animal model. (K) Tendon that was injured. (L) Injury model utilized. (M) EV delivery methodology. (N) Biomaterial used to supplement EVs.

Examining in vivo model selection, 34 studies (74%) used a rat model, 42 studies (91%) utilized young animals, and 41 studies (89%) used only males (Figure 2H–J). While there was a diversity of tendons selected for injury, Achilles tendon injuries were utilized in 24 studies (52%) (Figure 2K). In 29 studies (63%), tendons were injured via full transection followed by surgical repair, in six studies (13%) a partial transection and repair were performed, and 12 studies (26%) utilized a collagenase injection (Figure 2L). Thirty‐one studies (67%) delivered EVs a single time immediately after the injury (Figure 2M). Fourteen studies (30%) delivered EVs with a gelatin support matrix, four studies (9%) provided a collagen‐based support matrix, and 15 studies (33%) provided no biomaterial support (Figure 2N).

### 3.2 Exclusion Criteria, Blinding, Animal Housing Details, and Protocol Registration Were Poorly Reported

Next, we assessed reporting of included articles using the ARRIVE guidelines (Table S4). Study design, outcome measures, results, background, abstract, objectives, ethical statement, interpretation, data access, and conflicts of interest were adequately reported in the literature. Sample size, randomization, statistical methods, experimental animals, experimental procedures, and generalizability/translatability were unclear if adequately reported. Finally, inclusion/exclusion criteria, blinding, animal housing & husbandry, animal care & monitoring, and protocol registration were inadequately reported (Figure 3A). Examining the relationship between transparency of reporting and year of publication, we note that all included studies were published after 2018. In the infancy of this literature, there was no statistically significant relationship between transparency in reporting and time (Figure 3B).

**Figure 3:**
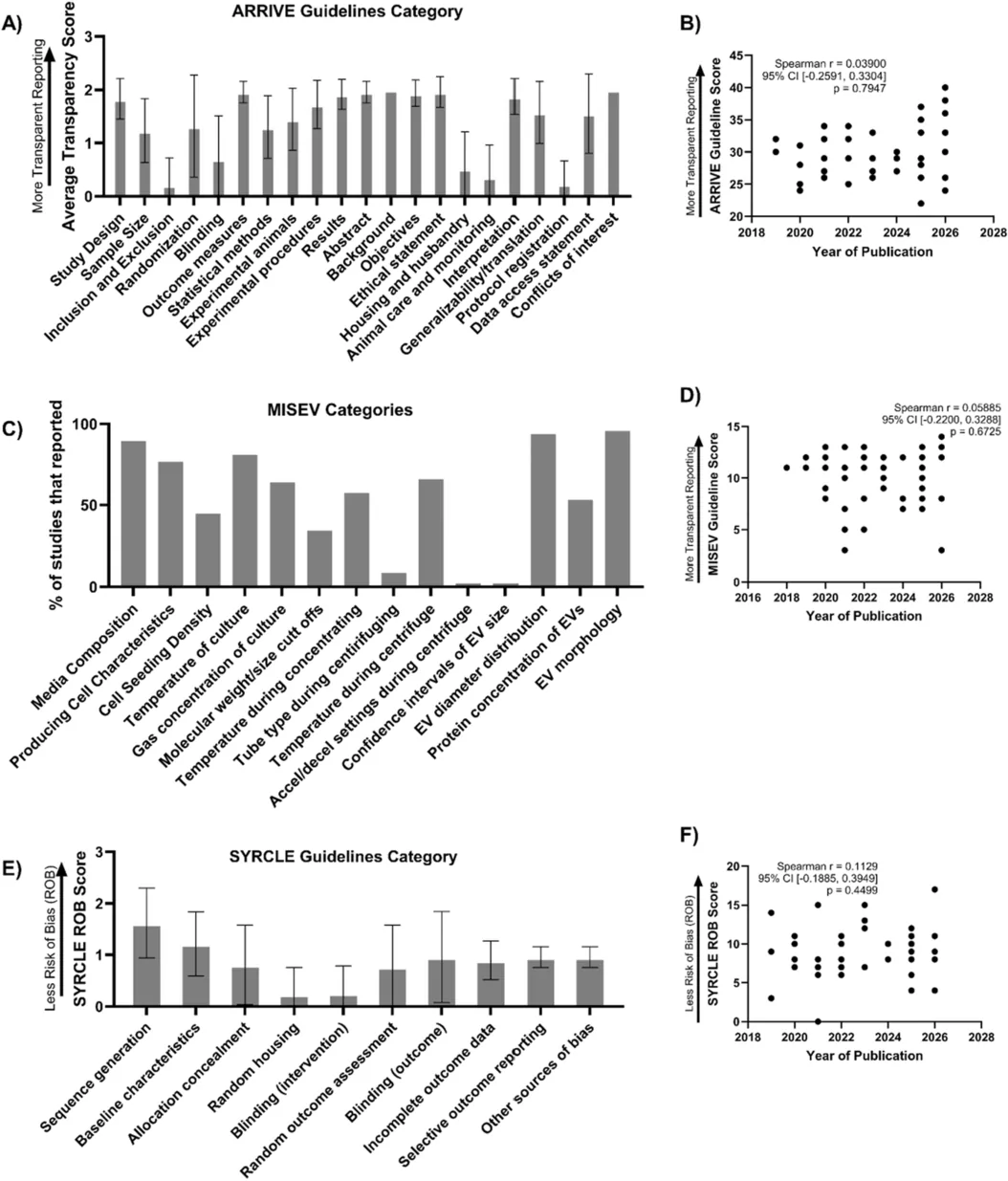
ARRIVE, MISEV, and SYRCLE ROB Reporting Guideline Scores. (A) ARRIVE guideline category assessment for included studies. (B) Relationship between year of publication and transparency in reporting, as assessed with the ARRIVE Guidelines. Table S4 includes individual score per paper. (C) 2018 MISEV guideline category assessment for included studies. (D) Relationship between year of publication and transparency in reporting, as assessed with the 2018 MISEV Guidelines. Table S5 includes individual score per paper as well as categories from the 2023 guidelines. (E) SYRCLE Risk of Bias category assessment for included studies. (F) Relationship between year of publication and risk of bias, as quantified by the SYRCLE criteria. Table S6 includes individual score per paper.

### 3.3 Several EV‐Specific Parameters Remain Poorly Reported in the Literature

Similarly, we also assessed the transparent reporting of EV characteristics using the MISEV guidelines. The 2018 MISEV Guidelines were used for analyses given all included manuscripts were published after 2018 (Table S5) [25, 35, 36]. When looking at the specific categories, we found most papers appropriately reported information on media composition, producing cell characteristics, culture temperature, gas concentration, temperature during concentrating, EV diameter distribution, and EV morphology. However, cell seeding density, molecular weight cut‐offs, tube type, settings during centrifugation, protein concentration in EVs, and confidence interval of EV size remain poorly reported (Figure 3C). We did not detect a statistically significant relationship between year of publication and adherence to the 2018 MISEV guidelines (Figure 3D).

### 3.4 The Risk of Bias in Publication in This Body of Literature Is Relatively High

When assessing adherence to SYRCLE ROB criteria, sequence generation was the only category consistently reported. Conversely, the remaining categories, including baseline characteristics, allocation concealment, random housing, blinding of intervention and outcome, random outcome assessment, incomplete outcome data, selective outcome reporting, and other sources of bias, had relatively high ROB (Figure 3E, Table S6). Moreover, we did not detect a statistically significant relationship between year of publication and risk of bias, as quantified by SYRCLE guidelines (Figure 3F).

### 3.5 EV Treatment Is Associated With Improved Histological Scores and Mechanical Properties of Tendon, Despite High Heterogeneity

Next, we performed meta‐analyses to assess the impact of EV treatment on histological score and mechanical properties. We found that EV treatment was associated with improved histological score when compared to tendons not treated with EVs (Figure 4A). Similarly, we found an association between improved mechanical properties and EV treatment. Specifically, the maximum load to failure (Figure 4B), tendon stiffness (Figure 4C), and Young's modulus (Figure 4D) increased in tendons treated with EVs relative to those that did not receive EV treatment. Interestingly, these differences were detected despite analyses exhibiting high heterogeneity (I^2^ > 90%). Using the demographic information from Figure 2, we attempted to perform subgroup analyses to identify the source of this heterogeneity, but unfortunately, given the high variability and the relatively small number of included studies (e.g., Figure 2F), we were unable to identify a statistically significant source (Table S7). Specifically, we did not detect an effect of sex, species, age, size, cell type or passage from which EVs were obtained; EV markers used for characterization; in vivo species, sex, age, tendon, model, biomaterial, time after injury or EV administration tissues (Table S7). Notably, we were unable to assess for an effect of native versus primed cells because there were not enough outcomes in both groups (i.e., only one study included in the meta‐analyses used primed cells). Additionally, for all of these analyses, funnel plots do not depict symmetrical spread across the inverted pyramid, which suggests publication bias, small‐study effects, or other sources of between‐study variation may be contributing to high heterogeneity (Figure S1). Given the high heterogeneity amongst the histological outcomes, we next sought to examine the relationship between dosage of EVs and effect size of histological outcome. Unfortunately, we were not powered to evaluate this relationship statistically, but we did visualize these relationships in Figure S2A–S2B.

**Figure 4:**
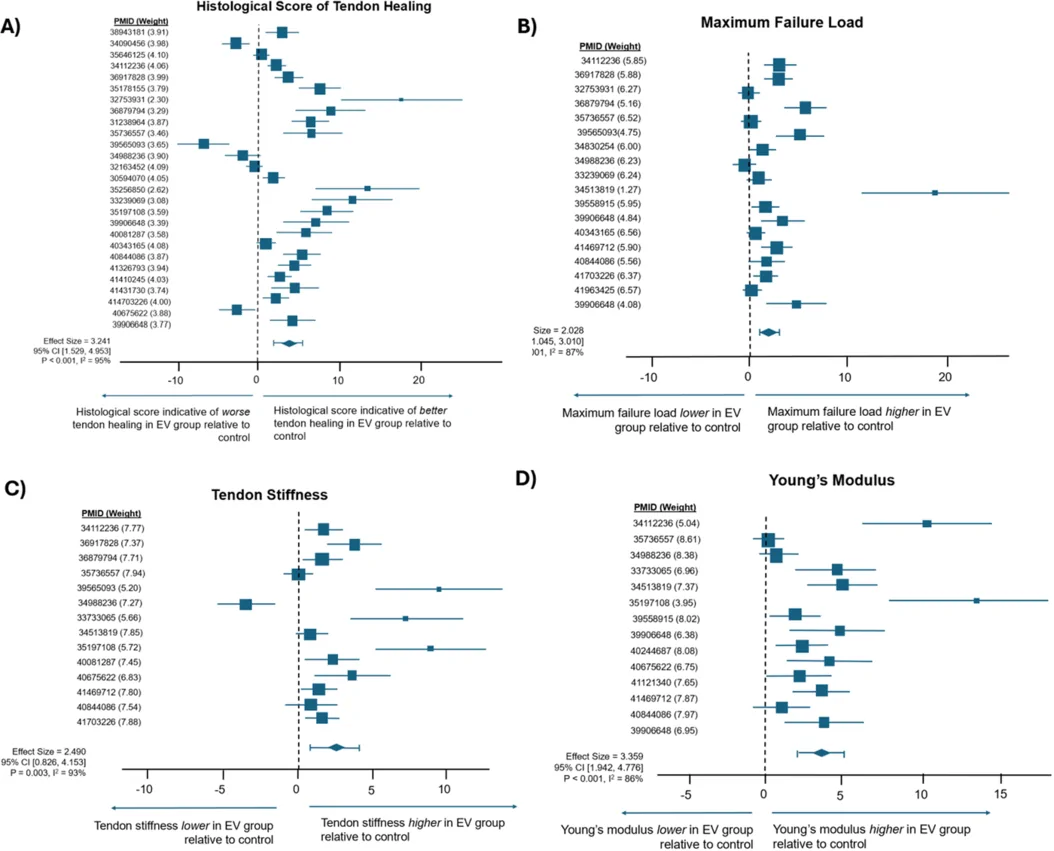
Meta analyses assessing the effects of EVs on histological score, load to failure, stiffness, and Young's modulus. (A) Meta‐analysis on histological scoring. (B) Meta‐analysis on maximum failure load. (C) Meta‐analysis on tendon stiffness. (D) Meta‐analysis on Young's modulus. Square = effect size for individual studies, with line indicating 95% confidence interval and size indicating the weight of the analysis. Diamond = average effect size from all included data.

### 3.6 EV Treatment Is Associated With Increased Tendon Type I Collagen Protein Expression and Type III Collagen Transcript Expression

Lastly, we performed a series of meta‐analyses to identify mechanisms that may be contributing to improvements in tendon histological structure and mechanical properties. We found that EV treatment was associated with increased protein expression of type I collagen but not type III collagen relative to untreated controls (Figure 5A–5B). Interestingly, our meta‐analysis revealed the opposite for transcript expression; specifically, EV treatment was associated with increased type III but not type I collagen transcript expression relative to untreated controls (Figure 5C–5D). We also detected a significant association between decreased IL‐6 protein expression and markers of senescence in EV treatment relative to untreated controls (Figure S2C–S2E). Again, funnel plots demonstrated a lack of symmetry across the inverted pyramid, suggesting other sources of variability may be contributing to these findings (Figure S3). As before, given high heterogeneity, we attempted to disentangle potential co‐drivers of this relationship but did not detect any significant interactions (Table S7).

**Figure 5:**
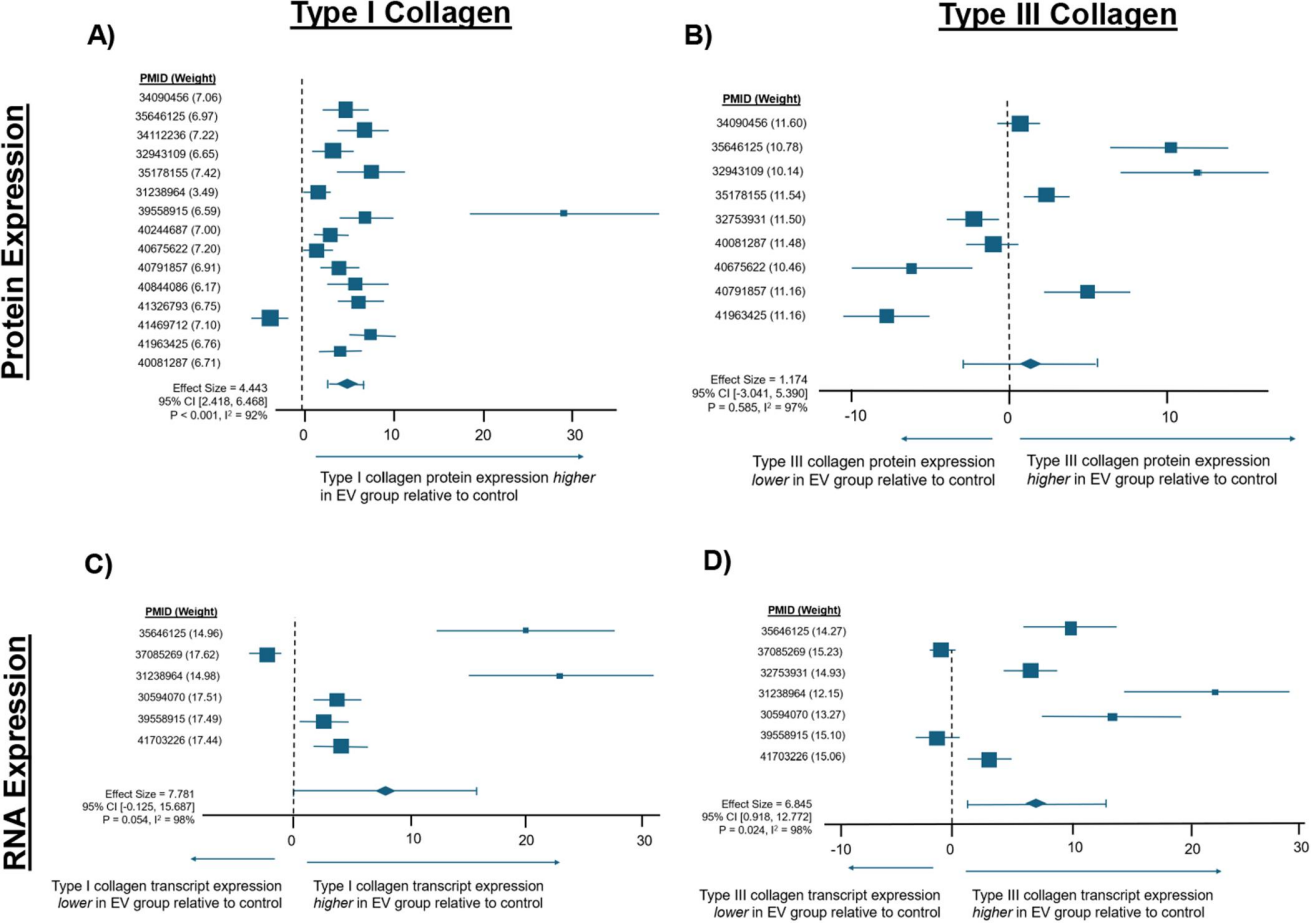
Meta analyses assessing the effects of EVs on type I and III collagen protein and transcript expression. (A) Meta‐analysis on type I collagen protein expression. (B) Meta‐analysis on type III collagen protein expression. (C) Meta‐analysis on type I collagen transcript expression. (D) Meta‐analysis on type III collagen transcript expression. Square = effect size for individual studies, with line indicating 95% confidence interval and size indicating the weight of the analysis. Diamond = average effect size from all included data.

## 4 Discussion

The purpose of this study was to systematically review the current body of peer‐reviewed literature to better understand the impact of EVs on tendon healing in pre‐clinical animal models. We found that most studies utilized young male animals, though a diversity of injury locations and types, EV dosage schemes, and biomaterials were implemented. Several features of rigor and reproducibility, such as exclusion criteria and blinding, as well as EV reporting guidelines, such as confidence interval of EV size, remain poorly reported in this body of literature, and risk‐of‐bias in publication is relatively high in this body of literature due to inadequate reporting. Despite high heterogeneity, meta‐analyses revealed that EV treatment was associated with improved histological evaluation of tendon healing and mechanical properties in preclinical models, including increased load to failure, tendon stiffness, and Young's modulus. EV treatment also increased type I collagen protein and type III collagen transcript expression, while decreasing protein expression of IL‐6 and markers of senescence.

Several studies have reported that tendon regeneration is mediated by age [85, 86] and sex [87, 88] and that EV effects on tissues are impacted by age and sex of the origin cells [89, 90]. Yet, these two variables are sparsely considered in this specific body of literature. The interacting effects of age and sex are particularly important given that post‐menopausal women have higher rates of tendon injuries than pre‐menopausal women and age‐matched men [91, 92], with documented effects of estrogens on tendon mechanical properties [93, 94]. Therefore, we recommend future studies integrate age‐by sex paradigms into their study design.

Although there was high heterogeneity in most of our meta‐analyses, we could not discern the origin of this heterogeneity. One area of particular interest is how dosage and frequency of treatment may impact outcome variables. To our surprise, few studies administered multiple doses of EVs and because of this, we were not able to quantitatively evaluate how this impacted outcomes. However, administering multiple doses over time may increase treatment efficacy, as several studies in different organ systems have supported a multi‐dose strategy [95, 96, 97]. We also visualized the relationship between dosage and effect size of histological outcomes due to not being statistically powered to formally analyze this relation and did not see any obvious patterns. It is unclear whether this is a true null finding or is due to the small sample sizes available in our analysis or other sources of variability in study designs. Nonetheless, EV dosage and frequency are two important variables that should be prioritized for targeted investigation in future studies.

One mechanistic insight gained from this study is the impact of EVs on type I and III collagen expression following tendon injury. In healthy tendons, approximately 95% of collagen is type I collagen [98]. Type III collagen expression, which has lower tensile strength, increases in the early stages following injury and is ultimately replaced by type I collagen, though this process can take years [99]. Our meta‐analyses were unable to discern a time‐based relationship between injury status and collagen turnover, likely due to variability among studies. Furthermore, while two studies in our review examined both transcript and protein expression of both type I and III collagen [42, 51], samples were all collected at the same time point, thus we were unable to evaluate how collagen transcripts and proteins change relative to each other across time. Understanding the relationship between collagen transcript and protein expression timing along the regenerative cascade is a critical area of clarification for future research to correctly interpret EV‐mediated effects on tissue repair.

Another question raised from our analyses is: are increases in collagen expression contributing productively to the regenerative cascade? Given that EV administration was also associated with improved mechanical properties and histological signs of healing, it would appear that increasing collagens is beneficial, but future mechanistic studies should consider the impact of EVs on collagen hydroxylation and further assess structure using techniques such as second harmonic generation or electron microscopy to ensure the newly deposited collagen is positively contributing to tendon structure and function.

Although this review adds important context to the growing body of literature investigating the potential therapeutic role of EVs on tendon healing, it does have some important limitations. Our review was not prospectively registered, and we excluded non‐English, non‐peer reviewed studies. The generalizability of these findings is limited to the demographics included in the studies (i.e., young, male tendons). Although this methodology is well established and was validated by a second author, all of the data utilized in our meta‐analysis were only extracted by a single author. Almost all of our meta‐analyses have high heterogeneity, and there are likely other variables impacting outcome variables that we could not identify. Specifically, the null meta‐analyses are challenging to interpret since we cannot identify if the study variability is contributing to the lack of null results or if they are true null results. We acknowledge that Hedges' g and a small‐sample size correction can reduce small‐sample bias which we did not use in our meta‐analysis due to the variability in sample size (small to moderate to large) and because our analysis used uncorrected SMDs. There is a reporting bias in the literature, with 33% not reporting the sex and 27% not reporting the age of the cells used to generate the cells, which may ultimately bias results. Additionally, for the protein and transcript meta‐analyses, we could not directly control for housekeeping protein/gene usage, which may have impacted results and makes pooling molecular outcomes with differing normalization metrics challenging. Given funnel plots demonstrated asymmetry, null findings or lower effect sizes may have been detected in other non‐published studies, and the lack of inclusion of this data may have affected meta‐analysis skew. Lastly, while we focused on in vivo studies, mechanistic insights can be gained from in vitro studies as well, as they enable isolation of specific cellular interactions, signaling pathways, and EV‐mediated effects under defined experimental conditions that are difficult to dissect in vivo.

## 5 Conclusions

The principal findings from this review are that EV treatment may be beneficial to tendon healing in preclinical models, as measured by histological outcome, load to failure, tendon stiffness, and Young's modulus. Mechanisms that may be contributing to these improved outcomes include EVs increasing type I collagen protein expression and type III collagen transcript expression, while decreasing protein expression of IL‐6 and senescence markers. Most published studies utilized young, male Achilles tendons. Further inclusion of aged and female animals will increase the translational potential of this body of literature. There is a significant amount of variability in injury type, dosage schema, and biomaterial utilization, and future studies should consider systematically testing the impact of these variables to discern their impact on EV effectiveness. Exclusion criteria, blinding, animal housing details, protocol registration, cell seeding density, gas concentration, tube type, and confidence interval of EV size remain poorly reported, and specific attention to more transparent reporting of these outcome variables should be included in future studies. Ultimately, these findings support further study of EV impacts on tendon healing and provide insights into how EVs may be enhancing healing.

## Author Contributions

All authors made substantial contributions in the following areas: (1) conception and design of the study, acquisition of data, analysis and interpretation of data, drafting of the article; (2) final approval of the article version to be submitted; and (3) agreement to be personally accountable for the author's own contributions and to ensure that questions related to the accuracy are appropriately investigated, resolved and the resolution documented in the literature. The specific contributions of the authors are as follows: Conceptualization: Allison C. Bean. Methodology: Gabrielle Gilmer. Formal analysis: Gabrielle Gilmer. Investigation: Gabrielle Gilmer, Kariman Shama, Liam Klingensmith, Jesse Zhang, Allison C. Bean. Resources: Allison C. Bean. Data curation: Gabrielle Gilmer, Kariman Shama, Liam Klingensmith, Jesse Zhang, Allison C. Bean. Visualization: Gabrielle Gilmer. Project administration: Gabrielle Gilmer, Allison C. Bean. Supervision: Allison C. Bean. Writing – original draft: Gabrielle Gilmer. Writing – review and editing: Gabrielle Gilmer, Kariman Shama, Liam Klingensmith, Jesse Zhang, Brittany Taylor Allison C. Bean.

## Conflicts of Interest

The authors declare no conflicts of interest.

## Supporting information


**Figure S1:** Funnel plots for meta‐analyses. (A) Histological scoring of tendon healing. (B) Load to failure. (C) Tendon stiffness. (D) Tendon Young's Modulus

**Figure S2:** The relationship between EV dose and histological outcome and meta‐analyses on IL‐6 and markers of senescence. (A) Relationship between EV dose and histological outcome, with EV dose quantified in µg. (B) Relationship between EV dose and histological outcome, with EV dose quantified in particles. (C) Meta‐analysis on IL‐6 protein expression. (D) Meta‐analysis on markers of senescence protein expression. (E) Funnel plot on IL‐6 protein expression. (F) Funnel plot of senescence protein expression

**Figure S3:** Funnel plots for meta‐analyses. Type I collagen (A) protein and (B) transcript. Type III collagen (C) protein and (D) transcript

Supporting File 1

Supporting File 2

Supporting File 3

## Data Availability

The data that supports the findings of this study are available in the Supporting material of this article.

## References

[1] Z. J. Li , Q. Q. Yang , and Y. L. Zhou , “Basic Research on Tendon Repair: Strategies, Evaluation, and Development,” Frontiers in Medicine 8 (2021): 664909. 10.3389/fmed.2021.664909 PMC8359775 34395467

[2] C. Chartier , H. ElHawary , A. Baradaran , J. Vorstenbosch , L. Xu , and J. I. Efanov , “Tendon: Principles of Healing and Repair,” Seminars in Plastic Surgery 35 (2021): 211–215. 10.1055/s-0041-1731632 PMC8432990 34526870

[3] J. A. Whitmore , J. G. Lyons , F. Berkay , and A. Minhas , “Epidemiology of Acute Primary Tendon Ruptures Presenting to Emergency Departments in the United States,” Orthopaedic Journal of Sports Medicine 13 (2025): 23259671251360402. 10.1177/23259671251360402 PMC12322374 40771883

[4] H. Minagawa , N. Yamamoto , H. Abe , et al., “Prevalence of Symptomatic and Asymptomatic Rotator Cuff Tears in the General Population: From Mass‐Screening in One Village,” Journal of Orthopaedics 10 (2013): 8–12. 10.1016/j.jor.2013.01.008 PMC3768248 24403741

[5] P. B. Voleti , M. R. Buckley , and L. J. Soslowsky , “Tendon Healing: Repair and Regeneration,” Annual Review of Biomedical Engineering 14 (2012): 47–71. 10.1146/annurev-bioeng-071811-150122 22809137

[6] B. M. Andres and G. A. C. Murrell , “Treatment of Tendinopathy: What Works, What Does Not, and What Is on the Horizon,” Clinical Orthopaedics & Related Research 466 (2008): 1539–1554. 10.1007/s11999-008-0260-1 PMC2505250 18446422

[7] M. Drummond Junior , C. Ayinon , M. Rodosky , D. Vyas , B. Lesniak , and A. Lin , “Predictive Factors for Failure of Conservative Management in the Treatment of Calcific Tendinitis of the Shoulder,” JSES International 5 (2021): 469–473. 10.1016/j.jseint.2021.01.013 PMC8178619 34136856

[8] N. A. Trasolini and B. R. Waterman , “Editorial Commentary: Rotator Cuff Repairs Fail at an Alarmingly High Rate During Long‐Term Follow‐Up: Graft Augmentation and Biologics May Improve Future Outcomes,” Arthroscopy: The Journal of Arthroscopic & Related Surgery: Official Publication of the Arthroscopy Association of North America and the International Arthroscopy Association 38 (2022): 2413–2416. 10.1016/j.arthro.2022.04.002 35940739

[9] A. Mandaleson , “Re‐Tears after Rotator Cuff Repair: Current Concepts Review,” Journal of Clinical Orthopaedics and Trauma 19 (2021): 168–174. 10.1016/j.jcot.2021.05.019 PMC8170498 34123722

[10] P. C. Wheeler , “Nearly Half of Patients With Chronic Tendinopathy May Have a Neuropathic Pain Component, With Significant Differences Seen Between Different Tendon Sites: A Prospective Cohort of More Than 300 Patients,” BMJ Open Sport & Exercise Medicine 8 (2022): e001297. 10.1136/bmjsem-2021-001297 PMC9301817 35965784

[11] O. O. Babatunde , J. Ensor , C. Littlewood , et al., “Comparative Effectiveness of Treatment Options for Subacromial Shoulder Conditions: A Systematic Review and Network Meta‐Analysis,” Therapeutic Advances in Musculoskeletal Disease 13 (2021): 1759720X211037530. 10.1177/1759720X211037530 PMC8435933 34527083

[12] J. Naunton , G. Street , C. Littlewood , T. Haines , and P. Malliaras , “Effectiveness of Progressive and Resisted and Non‐Progressive or Non‐Resisted Exercise in Rotator Cuff Related Shoulder Pain: A Systematic Review and Meta‐Analysis of Randomized Controlled Trials,” Clinical Rehabilitation 34 (2020): 1198–1216. 10.1177/0269215520934147 32571081

[13] X. Chen , I. A. Jones , C. Park , and C. T. Vangsness , “The Efficacy of Platelet‐Rich Plasma on Tendon and Ligament Healing: A Systematic Review and Meta‐Analysis With Bias Assessment,” American Journal of Sports Medicine 46 (2018): 2020–2032. 10.1177/0363546517743746 PMC6339617 29268037

[14] M. Vigano , G. Lugano , C. Perucca Orfei , et al., “Autologous Microfragmented Adipose Tissue Reduces the Catabolic and Fibrosis Response in an In Vitro Model of Tendon Cell Inflammation,” Stem Cells International 2019 (2019): 5620286. 10.1155/2019/5620286 PMC6915130 31885616

[15] C. D. Eliasberg and S. A. Rodeo , “Orthobiologics for Tendon Injuries,” Clinics in Sports Medicine 44 (2025): 749–762. 10.1016/j.csm.2024.10.006 41101908

[16] Y. J. Liu and C. Wang , “A Review of the Regulatory Mechanisms of Extracellular Vesicles‐Mediated Intercellular Communication,” Cell Communication and Signaling 21 (2023): 77. 10.1186/s12964-023-01103-6 PMC10100201 37055761

[17] A. Nagelkerke , M. Ojansivu , L. van der Koog , et al., “Extracellular Vesicles for Tissue Repair and Regeneration: Evidence, Challenges and Opportunities,” Advanced Drug Delivery Reviews 175 (2021): 113775. 10.1016/j.addr.2021.04.013 33872693

[18] J. Wu , Y. Piao , Q. Liu , and X. Yang , “Platelet‐Rich Plasma‐Derived Extracellular Vesicles: A Superior Alternative in Regenerative Medicine?,” Cell Proliferation 54 (2021): e13123. 10.1111/cpr.13123 PMC8666280 34609779

[19] E. Anitua , M. Troya , J. M. Falcon‐Pérez , S. López‐Sarrio , E. González , and M. H. Alkhraisat , “Advances in Platelet Rich Plasma‐Derived Extracellular Vesicles for Regenerative Medicine: A Systematic‐Narrative Review,” International Journal of Molecular Sciences 24 (2023): 13043. 10.3390/ijms241713043 PMC10488108 37685849

[20] S. Moghassemi , A. Dadashzadeh , M. J. Sousa , et al., “Extracellular Vesicles in Nanomedicine and Regenerative Medicine: A Review Over the Last Decade,” Bioactive Materials 36 (2024): 126–156. 10.1016/j.bioactmat.2024.02.021 PMC10915394 38450204

[21] M. Zou , J. Wang , and Z. Shao , “Therapeutic Potential of Exosomes in Tendon and Tendon‐Bone Healing: A Systematic Review of Preclinical Studies,” Journal of Functional Biomaterials 14 (2023): 299. 10.3390/jfb14060299 PMC10299056 37367263

[22] V. Kasula , V. Padala , N. Gupta , et al., “The Use of Extracellular Vesicles in Achilles Tendon Repair: A Systematic Review,” Biomedicines 12 (2024): 942. 10.3390/biomedicines12050942 PMC11117955 38790904

[23] H.‐M. Liu , M. Zhu , W. Ding , L. J. Ning , and T. W. Qin , “The Role of Extracellular Vesicles in the Treatment of Tendinopathy by Regulating the Pathological Immune Microenvironment,” Chemical Engineering Journal 499 (2024): 156489.

[24] V. Lu , M. Tennyson , J. Zhang , and W. Khan , “Mesenchymal Stem Cell‐Derived Extracellular Vesicles in Tendon and Ligament Repair—A Systematic Review of In Vivo Studies,” Cells 10 (2021): 2553. 10.3390/cells10102553 PMC8533909 34685532

[25] J. A. Welsh , D. C. I. Goberdhan , L. O'Driscoll , et al., “Minimal Information for Studies of Extracellular Vesicles (MISEV2023): From Basic to Advanced Approaches,” Journal of Extracellular Vesicles 13 (2024): e12404. 10.1002/jev2.12404 PMC10850029 38326288

[26] M. J. Page , J. E. McKenzie , P. M. Bossuyt , et al., “The PRISMA 2020 Statement: An Updated Guideline for Reporting Systematic Reviews,” BMJ 372 (2021): n71. 10.1136/bmj.n71 PMC8005924 33782057

[27] D. F. Stroup , “Meta‐Analysis of Observational Studies in Epidemiology: A Proposal for Reporting. Meta‐Analysis Of Observational Studies in Epidemiology (MOOSE) Group,” Journal of the American Medical Association 283 (2000): 2008–2012. 10.1001/jama.283.15.2008 10789670

[28] J. P. T. T. J. Higgins , J. Chandler , M. Cumpston , T. Li , M. J. Page , et al. 2022. Cochrane Handbook for Systematic Reviews of Interventions, 6.3 ed.

[29] H. M. Vesterinen , E. S. Sena , K. J. Egan , et al., “Meta‐Analysis of Data From Animal Studies: A Practical Guide,” Journal of Neuroscience Methods 221 (2014): 92–102. 10.1016/j.jneumeth.2013.09.010 24099992

[30] M. J. Page , L. Shamseer , and A. C. Tricco , “Registration of Systematic Reviews in PROSPERO: 30,000 Records and Counting,” Systematic Reviews 7 (2018): 32. 10.1186/s13643-018-0699-4 PMC5819709 29463298

[31] A. M. Methley , S. Campbell , C. Chew‐Graham , R. McNally , and S. Cheraghi‐Sohi , “PICO, PICOS and SPIDER: A Comparison Study of Specificity and Sensitivity in Three Search Tools for Qualitative Systematic Reviews,” BMC Health Services Research 14 (2014): 579. 10.1186/s12913-014-0579-0 PMC4310146 25413154

[32] D. Drevon , S. R. Fursa , and A. L. Malcolm , “Intercoder Reliability and Validity of WebPlotDigitizer in Extracting Graphed Data,” Behavior Modification 41 (2017): 323–339. 10.1177/0145445516673998 27760807

[33] G. Gilmer , A. C. Bean , H. Iijima , N. Jackson , R. C. Thurston , and F. Ambrosio , “Uncovering the ‘Riddle of Femininity’ in Osteoarthritis: A Systematic Review and Meta‐Analysis of Menopausal Animal Models and Mathematical Modeling of Estrogen Treatment,” Osteoarthritis and Cartilage 31 (2023): 447–457. 10.1016/j.joca.2022.12.009 PMC10033429 36621591

[34] N. Percie du Sert , V. Hurst , A. Ahluwalia , et al., “The ARRIVE Guidelines 2.0: Updated Guidelines for Reporting Animal Research,” PLoS Biology 18 (2020): e3000410. 10.1371/journal.pbio.3000410 PMC7360023 32663219

[35] J. Lotvall , A. F. Hill , F. Hochberg , et al., “Minimal Experimental Requirements for Definition of Extracellular Vesicles and Their Functions: A Position Statement From the International Society for Extracellular Vesicles,” Journal of Extracellular Vesicles 3 (2014): 26913. 10.3402/jev.v3.26913 PMC4275645 25536934

[36] C. Thery , K. W. Witwer , E. Aikawa , et al., “Minimal Information for Studies of Extracellular Vesicles 2018 (MISEV2018): A Position Statement of the International Society for Extracellular Vesicles and Update of the MISEV2014 Guidelines,” Journal of Extracellular Vesicles 7 (2018): 1535750. 10.1080/20013078.2018.1535750 PMC6322352 30637094

[37] C. R. Hooijmans , M. M. Rovers , R. B. de Vries, , M. Leenaars , M. Ritskes‐Hoitinga , and M. W. Langendam , “SYRCLE's Risk of Bias Tool for Animal Studies,” BMC Medical Research Methodology 14 (2014): 43. 10.1186/1471-2288-14-43 PMC4230647 24667063

[38] R. DerSimonian and N. Laird , “Meta‐Analysis in Clinical Trials,” Controlled Clinical Trials 7 (1986): 177–188. 10.1016/0197-2456(86)90046-2 3802833

[39] G. Wu , Q. Su , J. Li , et al., “NAMPT Encapsulated by Extracellular Vesicles From Young Adipose‐Derived Mesenchymal Stem Cells Treated Tendinopathy in a ‘One‐Stone‐Two‐Birds’ Manner,” Journal of Nanobiotechnology 21 (2023): 7. 10.1186/s12951-022-01763-5 PMC9814467 36604715

[40] D. Chen , Q. Tang , W. Song , and Y. He , “Platelet‐Derived Exosomes Alleviate Tendon Stem/Progenitor Cell Senescence and Ferroptosis by Regulating AMPK/Nrf2/GPX4 Signaling and Improve Tendon‐Bone Junction Regeneration in Rats,” Journal of Orthopaedic Surgery and Research 19 (2024): 382. 10.1186/s13018-024-04869-8 PMC11212425 38943181

[41] Z. Yao , J. Li , H. Xiong , et al., “MicroRNA Engineered Umbilical Cord Stem Cell‐Derived Exosomes Direct Tendon Regeneration by mTOR Signaling,” Journal of Nanobiotechnology 19 (2021): 169. 10.1186/s12951-021-00906-4 PMC8180131 34090456

[42] X. Zhang , A. Li , K. Han , et al., “Anti‐Inflammatory and Tendon‐Protective Effects of Adipose Stem Cell‐Derived Exosomes With Concomitant Use of Glucocorticoids,” Stem Cells International 2022 (2022): 1455226. 10.1155/2022/1455226 PMC9142315 35646125

[43] H. Shen and R. A. Lane , “Extracellular Vesicles From Primed Adipose‐Derived Stem Cells Enhance Achilles Tendon Repair by Reducing Inflammation and Promoting Intrinsic Healing,” Stem Cells 41 (2023): 617–627. 10.1093/stmcls/sxad032 PMC10267691 37085269

[44] H. Liu , M. Zhang , M. Shi , et al., “Adipose‐Derived Mesenchymal Stromal Cell‐Derived Exosomes Promote Tendon Healing by Activating Both SMAD1/5/9 and SMAD2/3,” Stem Cell Research & Therapy 12 (2021): 338. 10.1186/s13287-021-02410-w PMC8194238 34112236

[45] H. Shen , S. Yoneda , Y. Abu‐Amer , F. Guilak , and R. H. Gelberman , “Stem Cell‐Derived Extracellular Vesicles Attenuate the Early Inflammatory Response After Tendon Injury and Repair,” Journal of Orthopaedic Research 38 (2020): 117–127. 10.1002/jor.24406 PMC6917960 31286564

[46] M. Zhang , H. Liu , Q. Cui , et al., “Tendon Stem Cell‐Derived Exosomes Regulate Inflammation and Promote the High‐Quality Healing of Injured Tendon,” Stem Cell Research & Therapy 11 (2020): 402. 10.1186/s13287-020-01918-x PMC7499865 32943109

[47] J. Cai , J. Xu , Z. Ye , et al., “Exosomes Derived From Kartogenin‐Preconditioned Mesenchymal Stem Cells Promote Cartilage Formation and Collagen Maturation for Enthesis Regeneration in a Rat Model of Chronic Rotator Cuff Tear,” American Journal of Sports Medicine 51 (2023): 1267–1276. 10.1177/03635465231155927 36917828

[48] X. Li , Z. Su , K. Shen , et al., “Eugenol‐Preconditioned Mesenchymal Stem Cell‐Derived Extracellular Vesicles Promote Antioxidant Capacity of Tendon Stem Cells In Vitro and In Vivo,” Oxidative Medicine and Cellular Longevity 2022 (2022): 3945195. 10.1155/2022/3945195 PMC8847013 35178155

[49] Z. Yao , J. Li , X. Wang , et al., “MicroRNA‐21‐3p Engineered Umbilical Cord Stem Cell‐Derived Exosomes Inhibit Tendon Adhesion,” Journal of Inflammation Research 13 (2020): 303–316. 10.2147/JIR.S254879 PMC7354957 32753931

[50] X. Tong , Y. Xu , T. Zhang , et al., “Exosomes from CD133(+) Human Urine‐Derived Stem Cells Combined Adhesive Hydrogel Facilitate Rotator Cuff Healing by Mediating Bone Marrow Mesenchymal Stem Cells,” Journal of Orthopaedic Translation 39 (2023): 100–112. 10.1016/j.jot.2023.02.002 PMC9984782 36879794

[51] Z. Shi , Q. Wang , and D. Jiang , “Extracellular Vesicles From Bone Marrow‐Derived Multipotent Mesenchymal Stromal Cells Regulate Inflammation and Enhance Tendon Healing,” Journal of Translational Medicine 17 (2019): 211. 10.1186/s12967-019-1960-x PMC6593555 31238964

[52] X. Zhang , Z. Han , K. Han , et al., “Loading Mesenchymal Stem Cell‐Derived Exosomes Into a Traditionally Designed Rotator Cuff Patch: A Potential Strategy to Enhance the Repair of Chronic Rotator Cuff Tear Associated With Degenerative Changes,” American Journal of Sports Medicine 50 (2022): 2234–2246. 10.1177/03635465221096490 35736557

[53] Y. Wang , J. Z. Qin , C. Y. Xie , X. Z. Peng , J. H. Wang , and S. J. Wang , “Kartogenin‐Loaded Exosomes Derived From Bone Marrow Mesenchymal Stem Cells Enhance Chondrogenesis and Expedite Tendon Enthesis Healing in a Rat Model of Rotator Cuff Injury,” American Journal of Sports Medicine 52 (2024): 3520–3535. 10.1177/03635465241296141 39565093

[54] S. H. Chen , Z. Y. Chen , Y. H. Lin , et al., “Extracellular Vesicles of Adipose‐Derived Stem Cells Promote the Healing of Traumatized Achilles Tendons,” International Journal of Molecular Sciences 22 (2021): 12373. 10.3390/ijms222212373 PMC8618291 34830254

[55] E. P. Wellings , T. C. T. Huang , J. Li , et al., “Intrinsic Tendon Regeneration After Application of Purified Exosome Product: An In Vivo Study,” Orthopaedic Journal of Sports Medicine 9 (2021): 23259671211062929. 10.1177/23259671211062929 PMC8721391 34988236

[56] C. Gissi , A. Radeghieri , C. Antonetti Lamorgese Passeri , et al., “Extracellular Vesicles From Rat‐Bone‐Marrow Mesenchymal Stromal/Stem Cells Improve Tendon Repair in Rat Achilles Tendon Injury Model in Dose‐Dependent Manner: A Pilot Study,” PLoS One 15 (2020): e0229914. 10.1371/journal.pone.0229914 PMC7067391 32163452

[57] H. Cui , Y. He , S. Chen , D. Zhang , Y. Yu , and C. Fan , “Macrophage‐Derived miRNA‐Containing Exosomes Induce Peritendinous FibrosisAfter Tendon Injury Through the miR‐21‐5p/Smad7 Pathway,” Molecular Therapy–Nucleic Acids 14 (2019): 114–130. 10.1016/j.omtn.2018.11.006 PMC6307349 30594070

[58] Z. Zhu , R. Gao , T. Ye , et al., “The Therapeutic Effect of iMSC‐Derived Small Extracellular Vesicles on Tendinopathy Related Pain Through Alleviating Inflammation: An In Vivo and In Vitro Study,” Journal of Inflammation Research 15 (2022): 1421–1436. 10.2147/JIR.S345517 PMC8898180 35256850

[59] Y. Wang , G. He , Y. Guo , et al., “Exosomes From Tendon Stem Cells Promote Injury Tendon Healing Through Balancing Synthesis and Degradation of the Tendon Extracellular Matrix,” Journal of Cellular and Molecular Medicine 23 (2019): 5475–5485. 10.1111/jcmm.14430 PMC6653097 31148334

[60] S. C. Tao , J. Y. Huang , Z. X. Li , S. Zhan , and S. C. Guo , “Small Extracellular Vesicles With LncRNA H19 ‘Overload’: YAP Regulation as a Tendon Repair Therapeutic Tactic,” iScience 24 (2021): 102200. 10.1016/j.isci.2021.102200 PMC7937563 33733065

[61] J. Li , Z. Yao , H. Xiong , et al., “Extracellular Vesicles From Hydroxycamptothecin Primed Umbilical Cord Stem Cells Enhance Anti‐Adhesion Potential for Treatment of Tendon Injury,” Stem Cell Research & Therapy 11 (2020): 500. 10.1186/s13287-020-02016-8 PMC7687768 33239069

[62] Y. Yu , B. Sun , Z. Wang , et al., “Exosomes From M2 Macrophage Promote Peritendinous Fibrosis Posterior Tendon Injury via the MiR‐15b‐5p/FGF‐1/7/9 Pathway by Delivery of circRNA‐Ep400,” Frontiers in Cell and Developmental Biology 9 (2021): 595911. 10.3389/fcell.2021.595911 PMC8432299 34513819

[63] Q. Han , S. Wang , D. Chen , D. Gan , and T. Wang , “Exosomes Derived From Human Umbilical Cord Mesenchymal Stem Cells Reduce Tendon Injuries via the miR‐27b‐3p/ARHGAP5/RhoA Signaling Pathway,” Acta Biochimica et Biophysica Sinica 54 (2022): 232–242. 10.3724/abbs.2021026 PMC9909356 35130628

[64] K. Song , T. Jiang , P. Pan , Y. Yao , and Q. Jiang , “Exosomes From Tendon Derived Stem Cells Promote Tendon Repair Through miR‐144‐3p‐regulated Tenocyte Proliferation and Migration,” Stem Cell Research & Therapy 13 (2022): 80. 10.1186/s13287-022-02723-4 PMC8867681 35197108

[65] R. Chen , L. Ai , J. Zhang , and D. Jiang , “Dendritic Cell‐Derived Exosomes Promote Tendon Healing and Regulate Macrophage Polarization in Preventing Tendinopathy,” International Journal of Nanomedicine 19 (2024): 11701–11718. 10.2147/IJN.S466363 PMC11571930 39558915

[66] D. Li , S. Li , S. He , et al., “Restoring Tendon Microenvironment in Tendinopathy: Macrophage Modulation and Tendon Regeneration With Injectable Tendon Hydrogel and Tendon‐Derived Stem Cells Exosomes,” Bioactive Materials 47 (2025): 152–169. 10.1016/j.bioactmat.2025.01.016 PMC11791013 39906648

[67] L. Chunbo , L. Jia , Z. Jian , G. Yaxian , G. Chenwei , and X. Cong , “Exosomes Derived From Schwann Cells (SCDE) Promote Tendon Repair Through the PTEN/PI3K/Akt Signaling Pathway,” Journal of Orthopaedic Surgery and Research 20 (2025): 911. 10.1186/s13018-025-06352-4 PMC12539145 41121340

[68] X. Gao , Y. Chen , J. Wang , et al., “Mitochondria‐Rich Extracellular Vesicles From Bone Marrow Stem Cells Mitigate Muscle Degeneration in Rotator Cuff Tears in a Rat Model Through Macrophage M2 Phenotype Conversion,” Arthroscopy 41 (2025): 3487–3499. 10.1016/j.arthro.2025.03.033 40147598

[69] M. Hao , K. Xiang , K. Zhang , et al., “Bee Sting‐Shaped Microneedles for Accelerating Achilles Tendinopathy Healing via Enhanced Regulating Macrophage Polarization,” Journal of Nanobiotechnology 24 (2026): 492. 10.1186/s12951-026-04364-8 PMC13200503 41965678

[70] Y. D. Jeon , W. Yu , I. K. Shim , G. J. Moon , J. H. Choi , and K. H. Koh , “Regenerative Effect of Injectable Collagen Loaded With Mesenchymal Stem Cell‐Derived Extracellular Vesicles in a Collagenase‐Induced Tendinopathy Rat Model,” American Journal of Sports Medicine 54 (2026): 1088–1102. 10.1177/03635465261421555 41761488

[71] S. Kong , Y. Yang , Y. Gao , et al., “Biodegradable Cryo‐Self‐Assembled Silk Fibroin Sponge for Enzyme‐Responsive Exosome Delivery to Enhance Tendon Regeneration,” ACS Applied Bio Materials 8 (2025): 3697–3707. 10.1021/acsabm.4c01429 40244687

[72] J. Lin , H. Zhang , H. Fu , et al., “Bone Marrow Mesenchymal Stem Cell‐Derived Exosomes Enhance Tendon Regeneration and Promote TDSC Migration and Differentiation,” Cellular Signalling 144 (2026): 112515. 10.1016/j.cellsig.2026.112515 41932412

[73] K. Lin , X. Hu , J. Yan , R. Gao , S. Lin , and S. Zhou , “Exosomal miR‐212‐5p Promotes Tendon Repair via Targeting FOXO1 to Activate PP1A/YAP1 Signaling,” Communications Biology 8 (2025): 1798. 10.1038/s42003-025-09210-5 PMC12722425 41326793

[74] H. Liu , A. Zhang , M. Shi , et al., “The Treatment of Acute Tendon Injury With Small Extracellular Vesicles Originating From TNFAIP6(‐) ADSCs Subpopoulation Both In Vitro and In Vivo,” Stem Cell Research & Therapy 17 (2025): 61. 10.1186/s13287-025-04789-2 PMC12866520 41469712

[75] Y. Liu , Y. Pu , Q. Li , et al., “NSUN2‐Engineered Human Umbilical Cord Mesenchymal Stem Cell‐Derived Exosomes Ameliorate Tendon Injury by Promoting DNM2 Expression,” Regenerative Therapy 31 (2026): 101050. 10.1016/j.reth.2025.101050 PMC12768879 41503520

[76] M. Najeb , A. Samy , A. Rizk , E. Mosbah , I. Ibrahim , and G. Karrouf , “Mesenchymal Stem Cell Derived Exosomes and Injectable Platelet‐Rich Fibrin Enhance SDFT Healing in a Donkey Tendonitis Model,” Scientific Reports 16 (2026): 12013. 10.1038/s41598-026-44967-7 PMC13069002 41963425

[77] K. Peng , S. Wang , J. Li , et al., “BMSCs Loaded Exosome Hydrogel Promotes the Repair of Rotator Cuff Injury in Rats In Vivo,” Scientific Reports 16 (2026): 9447. 10.1038/s41598-026-40392-y PMC13005012 41703226

[78] Y. Wu , T. Zhang , Y. Miao , et al., “Hypoxia Induces HIF‐1α Activation in Tendon Stem Cells to Enhance Extracellular Vesicle‐Mediated Tendon Repair,” ACS Applied Materials & Interfaces 17 (2025): 42637–42657. 10.1021/acsami.5c05369 40675622

[79] Y. Xu , J. Huang , Y. Mai , et al., “CBD‐Conjugated BMP‐Inhibiting Exosomes on Collagen Scaffold Dual‐Target Achilles Tendon Repair: Synergistic Regeneration and Heterotopic Ossification Prevention,” Materials Today Bio 32 (2025): 101790. 10.1016/j.mtbio.2025.101790 PMC12059345 40343165

[80] Y. Xu , B. Wu , Z. Zhang , et al., “Exosome‐Infused DTM‐MPs Hydrogel Scaffold: A Smart Platform for Tendon Repair With Sustained Bioactivity and Anti‐Inflammatory Benefits,” ACS Nano 20 (2026): 1030–1046. 10.1021/acsnano.5c16275 41410245

[81] A. D. Zhang , H. C. Liu , T. T. Zhang , et al., “ADSC‐EVs Activate Autophagy via the AKT/FOXO1 Pathway to Inhibit Tendon Fibrosis,” American Journal of Sports Medicine 53 (2025): 2618–2632. 10.1177/03635465251358465 40844086

[82] A. Zhang , Q. Xie , S. Guo , et al., “Platelet‐Rich Plasma‐Derived Exosomes Have the Novel Ability to Alleviate Insertional Achilles Tendinopathy by Promoting Tenogenesis in Tendon Stem/Progenitor Cells,” Biomaterials Advances 173 (2025): 214272. 10.1016/j.bioadv.2025.214272 40081287

[83] R. Zhang , H. Li , Y. Mu , et al., “Turmeric‐Derived Extracellular Vesicles Loaded Microneedle System Attenuates Rotator Cuff Degeneration by Orchestrating Energetic Metabolism,” Materials Today Bio 35 (2025): 102590. 10.1016/j.mtbio.2025.102590 PMC12718207 41431730

[84] Y. Zhang , R. Zhang , T. Zhang , et al., “Restoration of Tendon Repair Microenvironment by Grapefruit Exosome‐Loaded Microneedle System for Tendinopathy Therapy,” Frontiers in Bioengineering and Biotechnology 13 (2025): 1615650. 10.3389/fbioe.2025.1615650 PMC12336195 40791857

[85] K. Y. C. Kwan , K. W. K. Ng , Y. Rao , et al., “Effect of Aging on Tendon Biology, Biomechanics and Implications for Treatment Approaches,” International Journal of Molecular Sciences 24 (2023): 15183. 10.3390/ijms242015183 PMC10607611 37894875

[86] J. E. Ackerman , I. Bah , J. H. Jonason , M. R. Buckley , and A. E. Loiselle , “Aging Does Not Alter Tendon Mechanical Properties During Homeostasis, but Does Impair Flexor Tendon Healing,” Journal of Orthopaedic Research 35 (2017): 2716–2724. 10.1002/jor.23580 PMC5645212 28419543

[87] D. C. Sarver , Y. A. Kharaz , K. B. Sugg , J. P. Gumucio , E. Comerford , and C. L. Mendias , “Sex Differences in Tendon Structure and Function,” Journal of Orthopaedic Research 35 (2017): 2117–2126. 10.1002/jor.23516 PMC5503813 28071813

[88] J. C. Marvin , M. E. Brakewood , M. L. S. Poon , and N. Andarawis‐Puri , “Regenerative MRL/MpJ Tendon Cells Exhibit Sex Differences in Morphology, Proliferation, Mechanosensitivity, and Cell‐ECM Organization,” Journal of Orthopaedic Research 41 (2023): 2273–2286. 10.1002/jor.25562 37004178

[89] N. Noren Hooten , A. M. Byappanahalli , M. Vannoy , V. Omoniyi , and M. K. Evans , “Influences of Age, Race, and Sex on Extracellular Vesicle Characteristics,” Theranostics 12 (2022): 4459–4476. 10.7150/thno.72676 PMC9169362 35673574

[90] J. Burrello , J. Goi , A. Burrello , et al., “Age‐ and Sex‐Related Variations in Extracellular Vesicle Profiling for the Assessment of Cardiovascular Risk: The EVaging Index,” NPJ Aging 10 (2024): 63. 10.1038/s41514-024-00189-7 PMC11659617 39702460

[91] M. Abate , C. Schiavone , L. Di Carlo , and V. Salini , “Prevalence of and Risk Factors for Asymptomatic Rotator Cuff Tears in Postmenopausal Women,” Menopause 21 (2014): 275–280. 10.1097/GME.0b013e31829638e3 23760436

[92] S. S. Khadilkar , “Musculoskeletal Disorders and Menopause,” Journal of Obstetrics and Gynecology of India 69 (2019): 99–103. 10.1007/s13224-019-01213-7 PMC6430266 30956461

[93] A. Frizziero , F. Vittadini , G. Gasparre , et al., “Impact of Oestrogen Deficiency and Aging on Tendon: Concise Review,” Muscles, Ligaments and Tendons Journal 4 (2014): 324–328. PMC4241423 25489550

[94] D. R. Leblanc , M. Schneider , P. Angele , G. Vollmer , and D. Docheva , “The Effect of Estrogen on Tendon and Ligament Metabolism and Function,” Journal of Steroid Biochemistry and Molecular Biology 172 (2017): 106–116. 10.1016/j.jsbmb.2017.06.008 28629994

[95] M. Wysoczynki , A. Khan , and R. Bolli , “New Paradigms in Cell Therapy: Repeated Dosing, Intravenous Delivery, Immunomodulatory Actions, and New Cell Types,” Circulation Research 123 (2018): 138–158. 10.1161/CIRCRESAHA.118.313251 PMC6050028 29976684

[96] T. L. Kennedy , A. J. Russell , and P. Riley , “Experimental Limitations of Extracellular Vesicle‐Based Therapies for the Treatment of Myocardial Infarction,” Trends in Cardiovascular Medicine 31 (2021): 405–415. 10.1016/j.tcm.2020.08.003 PMC8501308 32822840

[97] D. W. Hagey , M. Ojansivu , B. R. Bostancioglu , et al., “The Cellular Response to Extracellular Vesicles Is Dependent on Their Cell Source and Dose,” Science Advances 9 (2023): eadh1168. 10.1126/sciadv.adh1168 PMC11629882 37656796

[98] K. M. Heinemeier , J. L. Olesen , F. Haddad , et al., “Expression of Collagen and Related Growth Factors in Rat Tendon and Skeletal Muscle in Response to Specific Contraction Types,” Journal of Physiology 582 (2007): 1303–1316. 10.1113/jphysiol.2007.127639 PMC2075262 17540706

[99] L. A. Dahlgren , B. D. Brower‐Toland , and A. J. Nixon , “Cloning and Expression of Type III Collagen in Normal and Injured Tendons of Horses,” American Journal of Veterinary Research 66 (2005): 266–270. 10.2460/ajvr.2005.66.266 15757126

