# Supplementary material for: Extracellular Vesicles Are Associated With Biological, Mechanical, and Structural Tendon Healing Metrics in Preclinical Models: A Systematic Review and Meta‐Analysis
Source: J Orthop Res. 2026 Oct 8;44(10):e70289. doi: 10.1002/jor.70289 (PMC13647102; doi:10.1002/jor.70289)
Supplement: Supplementary file 2 [file JOR-44-0-s002.pdf]

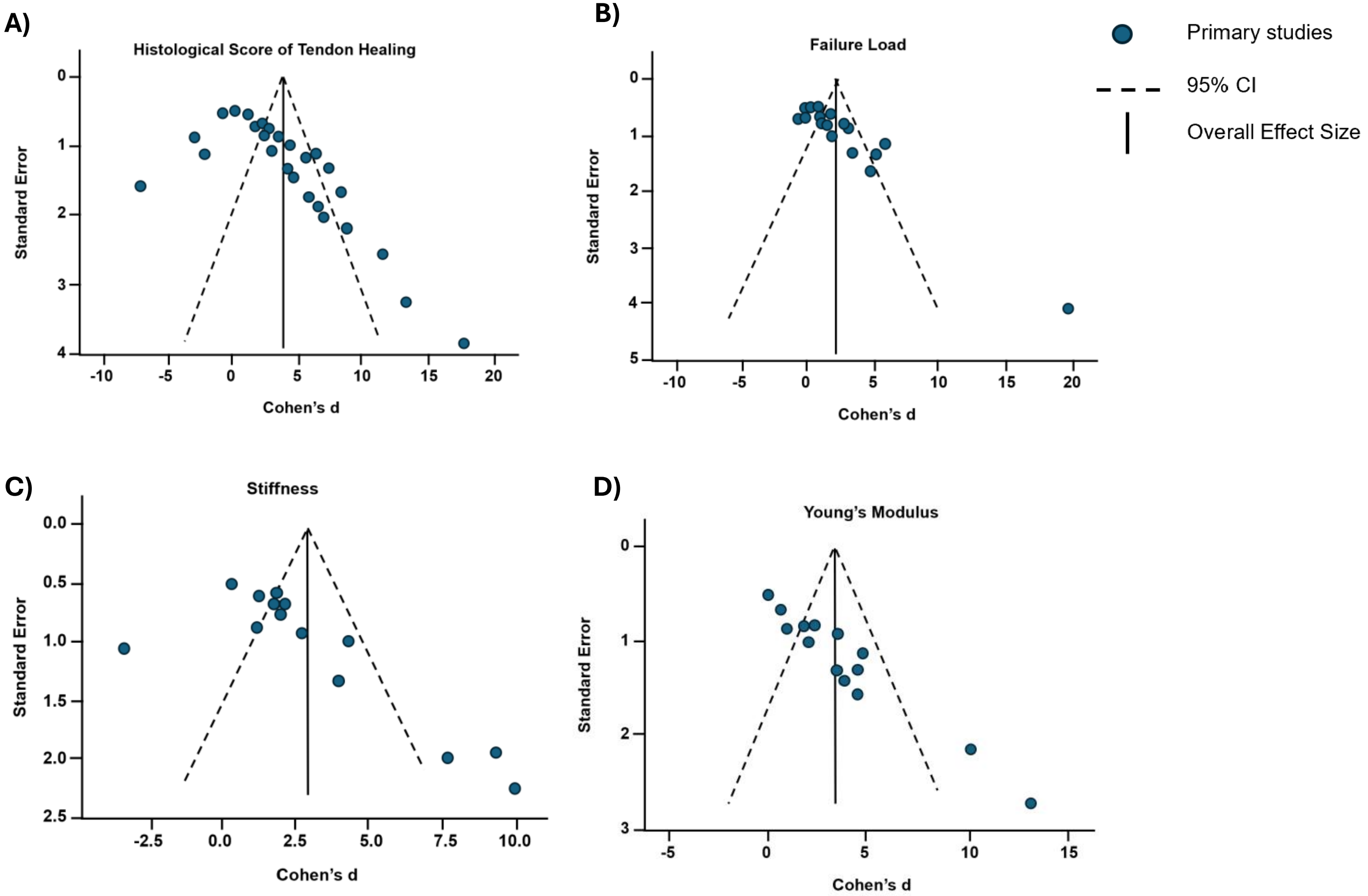

A)
Histological Score of Tendon Healing
Standard Error
0
1
2
3
4
-10
-5
0
5
10
15
20
Cohen's d
B)
Failure Load
Standard Error
0
1
2
3
4
5
-10
-5
0
5
10
15
20
Cohen's d
Primary studies
95% CI
Overall Effect Size
C)
Stiffness
Standard Error
0.0
0.5
1.0
1.5
2.0
2.5
-2.5
0.0
2.5
5.0
7.5
10.0
Cohen's d
D)
Young's Modulus
Standard Error
0
1
2
3
-5
0
5
10
15
Cohen's d
