# Supplementary material for: Extracellular Vesicles Are Associated With Biological, Mechanical, and Structural Tendon Healing Metrics in Preclinical Models: A Systematic Review and Meta‐Analysis
Source: J Orthop Res. 2026 Oct 8;44(10):e70289. doi: 10.1002/jor.70289 (PMC13647102; doi:10.1002/jor.70289)
Supplement: Supplementary file 3 [file JOR-44-0-s003.pdf]

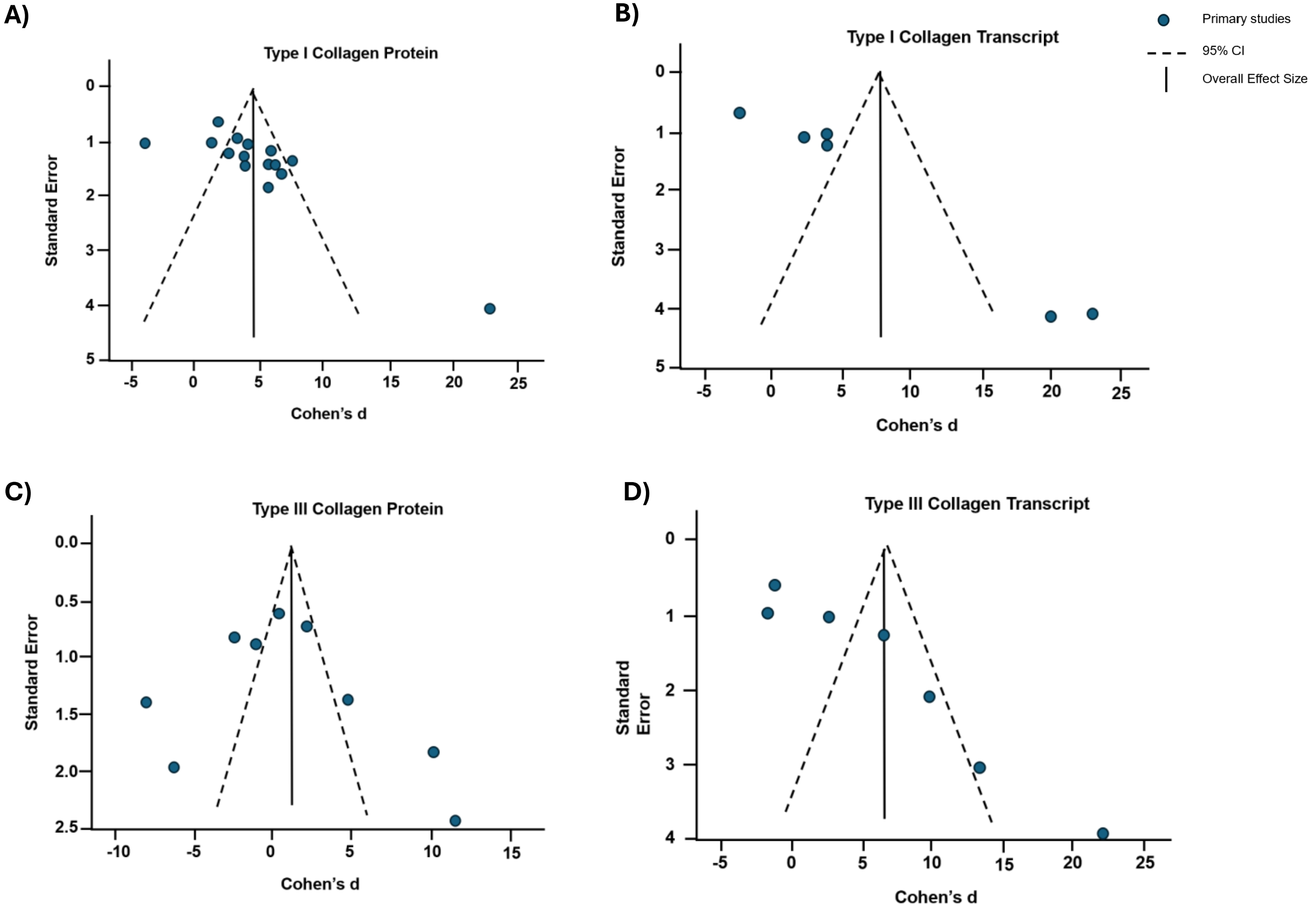
A)
Type I Collagen Protein
Standard Error
Cohen's d
B)
Type I Collagen Transcript
Primary studies
95% CI
Overall Effect Size
C)
Type III Collagen Protein
D)
Type III Collagen Transcript
