# Supplementary material for: Extracellular Vesicles Are Associated With Biological, Mechanical, and Structural Tendon Healing Metrics in Preclinical Models: A Systematic Review and Meta‐Analysis
Source: J Orthop Res. 2026 Oct 8;44(10):e70289. doi: 10.1002/jor.70289 (PMC13647102; doi:10.1002/jor.70289)
Supplement: Supplementary file 4 [file JOR-44-0-s004.pdf]

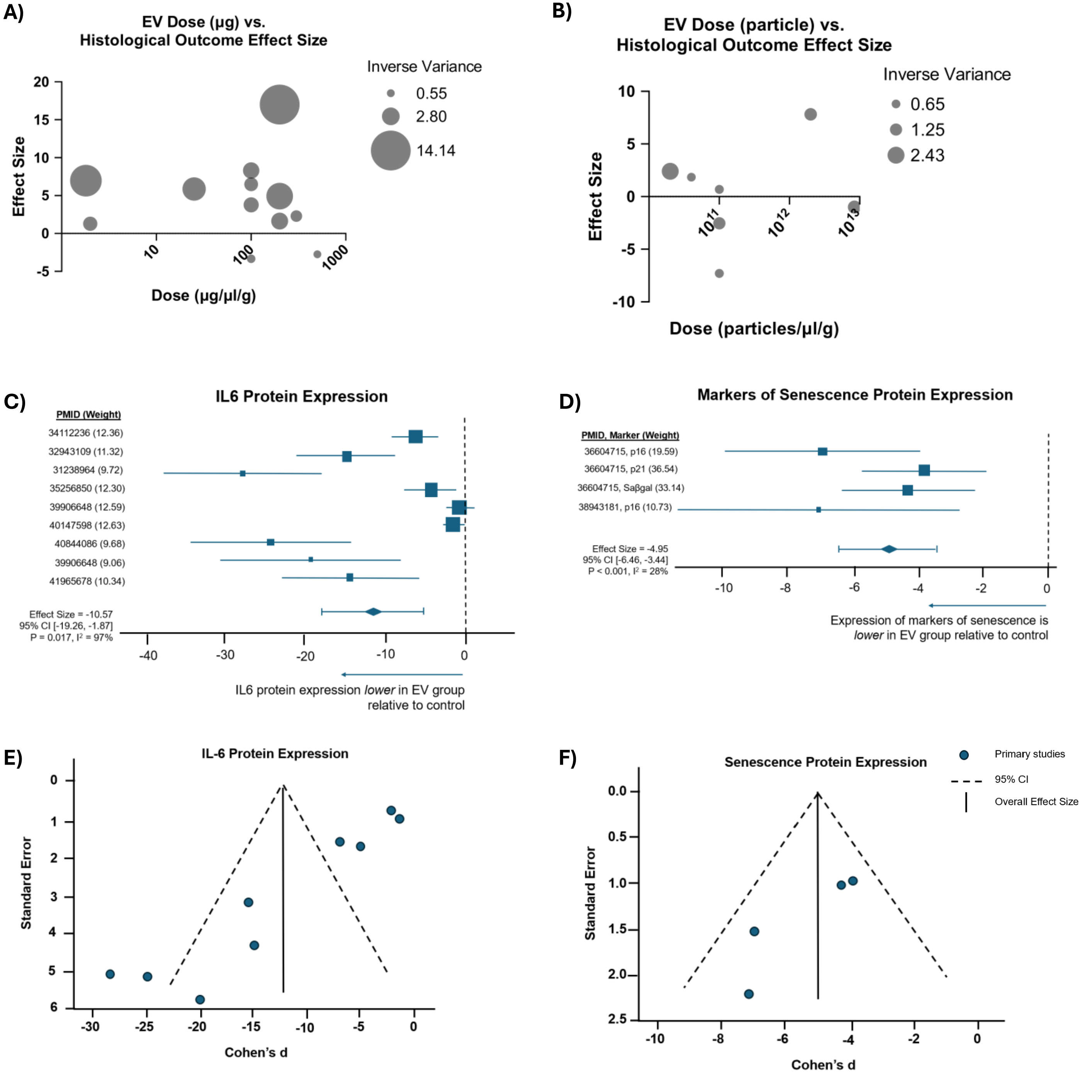
A)
EV Dose (µg) vs.
Histological Outcome Effect Size
Inverse Variance
0.55
2.80
14.14
Effect Size
20
15
10
5
0
-5
10
100
1000
Dose (µg/µl/g)
B)
EV Dose (particle) vs.
Histological Outcome Effect Size
Inverse Variance
0.65
1.25
2.43
Effect Size
10
5
0
-5
-10
$10^{11}$
$10^{12}$
$10^{13}$
Dose (particles/µl/g)
C)
IL6 Protein Expression
PMID (Weight)
34112236 (12.36)
32943109 (11.32)
31238964 (9.72)
35256850 (12.30)
39906648 (12.59)
40147598 (12.63)
40844086 (9.68)
39906648 (9.06)
41965678 (10.34)
Effect Size = -10.57
95% CI [-19.26, -1.87]
P = 0.017, I² = 97%
-40
-30
-20
-10
0
IL6 protein expression lower in EV group
relative to control
D)
Markers of Senescence Protein Expression
PMID, Marker (Weight)
36604715, p16 (19.59)
36604715, p21 (36.54)
36604715, Saβgal (33.14)
38943181, p16 (10.73)
Effect Size = -4.95
95% CI [-6.46, -3.44]
P < 0.001, I² = 28%
-10
-8
-6
-4
-2
0
Expression of markers of senescence is
lower in EV group relative to control
E)
IL-6 Protein Expression
Standard Error
0
1
2
3
4
5
6
-30
-25
-20
-15
-10
-5
0
Cohen's d
F)
Senescence Protein Expression
Primary studies
95% CI
Overall Effect Size
Standard Error
0.0
0.5
1.0
1.5
2.0
2.5
-10
-8
-6
-4
-2
0
Cohen's d
